# A meta-analysis of the effect of antibody therapy for the prevention of severe respiratory syncytial virus infection

**DOI:** 10.1186/1471-2334-9-106

**Published:** 2009-07-05

**Authors:** Shaun K Morris, Biljana Dzolganovski, Joseph Beyene, Lillian Sung

**Affiliations:** 1 Pediatric Infectious Diseases, Hospital for Sick Children, University of Toronto, Toronto, ON, Canada; 2 Child Health Evaluative Sciences Hospital for Sick Children, Toronto, ON, Canada; 3 Division of Haematology/Oncology Hospital for Sick Children, University of Toronto, Toronto, ON, Canada

## Abstract

**Background:**

The primary objective of this meta-analytic study was to determine the impact of RSV-IGIV and palivizumab on risk of respiratory syncytial virus (RSV)-related hospitalization. Secondary objectives were to determine if antibody therapy decreases the risk of RSV infection, intensive care admission, mechanical ventilation, and mortality in high risk infant populations.

**Methods:**

We performed searches of electronic data bases from 1966 to April 2009. Inclusion and exclusion criteria were defined *a priori*. Inclusion criteria were as follows: 1) There was randomization between polyclonal or monoclonal antibodies and placebo or no therapy, and 2) Polyclonal or monoclonal antibodies were given as prophylaxis.

**Results:**

Of the six included studies, three utilized RSV-IGIV (total of 533 randomized to treatment groups) and three utilized palivizumab (total of 1,663 randomized to treatment groups). The absolute risk of hospitalization in the control arms was 12% and overall RR for all 2,196 children who received one of the antibody products was 0.53 (95% CI 0.43, 0.66), P < 0.00001. When looking only at the children who received palivizumab, the RR for hospitalization was 0.50 (95% CI 0.38, 0.66), P < 0.00001. For the children receiving RSV-IGIV, the RR for hospitalization was 0.59 (95% CI 0.42, 0.83, P < 0.002). The use of palivizumab resulted in a significant decrease in admission to the ICU (RR 0.29 (95% CI 0.14, 0.59; P = 0.0007). There was no significant reduction in the risk of mechanical ventilation or mortality with the use of antibody prophylaxis. Infants born at less than 35 weeks gestational age, and those with chronic lung and congenital heart disease all had a significant reduction in the risk of RSV hospitalization with children born under 35 weeks gestational age showing a trend towards the greatest benefit.

**Conclusion:**

Both palivizumab and RSV-IGIV decrease the incidence of RSV hospitalization and ICU admission and their effect appears to be qualitatively similarly. There was neither a statistically significant reduction in the incidence of mechanical ventilation nor in all cause mortality. This meta-analysis separately quantifies the impact of RSV-IGIV and palivizumab on various measures of severe RSV disease and builds upon a previous study that was only able to examine the pooled effect of all antibody products together.

## Background

Respiratory syncytial virus (RSV) is a ubiquitous enveloped RNA paramyxovirus. Two strains, subtypes A and B, have been identified and often circulate concurrently in annual epidemics. In temperate climates, peak incidence occurs in the winter and early spring months. RSV is the most important cause of bronchiolitis and viral pneumonia in infants and young children. Over half of all infants in the United States are infected in their first year of life and nearly 100% have been infected by the age of two [1]. Humans are the only known reservoir for RSV and transmission is via direct or close contact with contaminated secretions.

Most healthy term infants do not require hospitalization as a result of RSV infection and mortality in these infants is less than 1%. However infants who are pre-term or have underlying chronic conditions including chronic lung disease (CLD) or congenital heart disease (CHD) are at higher risk for severe disease. Hospitalization and mortality in these higher risk groups is thought to be approximately 10% and 3% respectively [2,3].

The absence of a vaccine against RSV infection led to studies examining the effectiveness of passive antibody preparations. Two RSV passive antibody preparations were originally licensed. RSV immune globulin (RSV-IGIV) (RespiGam, MedImmune, Gaithersburg, MD), an intravenously administered immune globulin product derived from pooled adult human plasma selected for high titers of neutralizing antibody against RSV, was approved by the United States Federal Drug Administration (FDA) in January 1996 for use in infants and children younger than 24 months with CLD or a history of premature birth (< 35 weeks of gestation). In mid 1998, the FDA approved the use of palivizumab (Synagis, MedImmune, Gaithersburg, MD), a humanized monoclonal antibody directed against the RSV fusion protein, for the reduction of severe lower respiratory tract RSV infection in high risk infants and children. Palivizumab is administered intramuscularly on a monthly basis during the RSV season.

Single randomized trials have consistently showed that prophylaxis with RSV-IGIV and palivizumab can reduce hospitalization. However, these trials were not powered to examine more rare outcomes such as need for intensive care admission, need for mechanical ventilation, and mortality, nor were they powered for subgroup (premature, CLD, CHD) analysis. A previous meta-analysis has been published that concluded that RSV-IGIV and palivizimab significantly decreased the incidence of hospitalization and intensive care admission due to RSV infection but had no effect on incidence of mechanical ventilation or mortality [4]. However, this meta-analysis only included one study of palivizumab and was thus unable to examine the effect of the two antibody products. Additional randomized trials of palivizumab have been published since the original meta-analysis. We hypothesized that updating this meta-analysis and combining results of all randomized trials may be able to demonstrate whether prophylaxis with these agents can reduce severity of disease in high risk populations (i.e. premature infants and infants with CHD). Therefore, our primary objective was to determine the impact of RSV-IGIV and palivizumab on risk of RSV-related hospitalization. As the primary randomized controlled studies were not powered to show effect on other measures of severe RSV disease, our secondary objectives were to determine if antibody therapy decreases the risk of RSV infection, intensive care admission, mechanical ventilation, and mortality in high risk populations.

## Methods

### Data Sources and Searches

We developed a protocol for the review and followed standard QUOROM reporting guidelines [5]. We performed electronic searches of Ovid MEDLINE from 1966 to the end of July 2008, of EMBASE from 1980 to the end of April 2009, and of the Cochrane Central Register of Controlled Trials until the first quarter of 2009. The search strategy included the following Medical Subject Heading terms and text words: respiratory syncytial virus, bronchiolitis, prophylaxis, prophylactic, prevention, passive immunization, RSVIG, synagis and palivizumab. The search was limited to studies in humans and controlled trials and meta-analyses. As this study is a meta-analysis of primary studies, no specific ethical approval is required.

### Study Selection

Inclusion and exclusion criteria were defined *a priori*. Inclusion criteria were as follows: 1) There was randomization between polyclonal or monoclonal antibodies and placebo or no therapy, and 2) Polyclonal or monoclonal antibodies were given as prophylaxis. For describing reasons for exclusion, we used a hierarchical system in which reasons for exclusion were ranked in the following order: 1) Absence of placebo or no treatment arm, 2) Allocation not randomized, 3) Polyclonal or monoclonal antibody given as treatment rather than as prophylaxis, and 4) Duplicate publication. There was no language restriction for inclusion in this meta-analysis.

Two reviewers (SM and BD) independently evaluated titles and abstracts of publications identified by the search strategy, and any potentially relevant publication was retrieved in full. The reviewers were not blinded to study authors or outcomes. Final inclusion of studies into the meta-analysis was by agreement of both reviewers. Disagreement was adjudicated by a third author, LS. Agreement between reviewers on inclusion was evaluated using a kappa statistic. Strength of agreement as evaluated by the kappa statistic was defined as slight (0.00–0.20), fair (0.21–0.40), moderate (0.41–0.60), substantial (0.61–0.80) or almost perfect (0.81–1.00) [6].

### Data Extraction and Quality Assessment

The outcome measures were chosen to be representative of severe RSV lower respiratory tract disease. These include the primary outcome measure of hospitalization. Secondary outcome measures were the risk of RSV infection, and among those with RSV disease, the risk of ICU admission, mechanical ventilation, and mortality. Other outcome measures included days of hospitalization, days of intensive care unit admission, and days of mechanical ventilation. In studies that used multiple doses of antibody product in different arms of the study, only data for recipients of 750 mg/kg of RSV-IGIV or 15 mg/kg of palivizumab, the universally used dosages in clinical practice, were abstracted. Two authors (SM and BD) independently abstracted all data to standardized data collection forms.

Study quality was assessed using the Jadad scale [7]. The Jadad scale was designed to examine elements of clinical studies that may affect bias. The scale ranges from 0 to 5, with a higher score reflecting higher quality, and examines the adequacy of randomization, double-blinding, and descriptions of withdrawals and dropouts.

### Statistical Methods

This meta-analysis combined data at the study level and not at the individual patient level. Outcome data were synthesized using relative risk (RR) as the effect measure; RR greater than 1.0 suggests that an intervention is associated with an increase in that outcome and RR less than 1.0 suggests a decrease in that outcome. Effect sizes were weighted by the inverse of the variance.

Because we anticipated heterogeneity between studies, a random effects model was used for all analyses [8]. Subgroup analyses were performed for infants and children born prematurely, with CLD, and with CHD for the outcome of RSV hospitalization. There were insufficient events to perform subgroup analyses for other outcomes.

We tested for heterogeneity using the Cochran Q test and quantified the degree of heterogeneity with the I^2 ^statistic. The I^2 ^statistic ranges from 0–100% and measures the degree of inconsistency across studies in a meta-analysis as low, moderate, and high to I^2 ^values of 25%, 50%, and 75%, respectively [9].

Publication bias, which occurs when small studies are published only if the results are positive, was examined using a funnel plot. This plot displays RR on the x-axis and the inverse of variance of the effect on the y-axis. Asymmetry, without studies in the bottom left or right corner, depending on the effect measure, suggests publication bias. In the event of possible publication bias, the 'trim and fill' technique was used to determine the impact of such bias [10]. With this technique, outlying studies are deleted and hypothetical negative studies with equal weight are created to determine the robustness of the conclusions of the analysis. This meta-analysis was performed using Review manager (RevMan) (Version 4.2, The Cochrane Collaboration, Oxford, England).

## Results

### Study Selection and Characteristics

**Figure 1:**
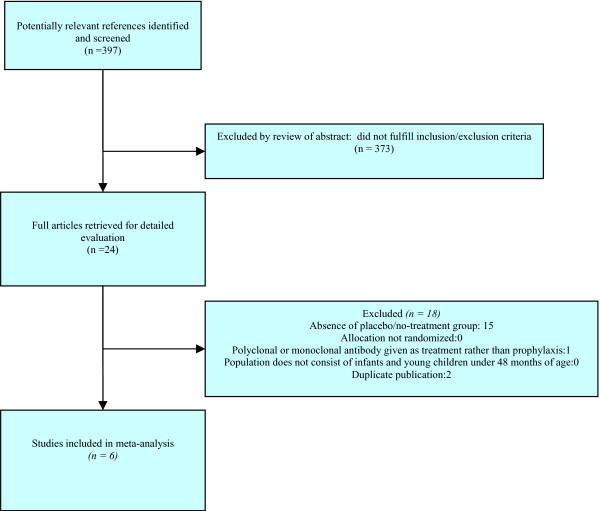
**Flow diagram of trial identification and selection**.

Figure 1 demonstrates the flow of trial identification and selection. The original search produced a total of 1,000 articles. Following initial review of titles, 397 potentially relevant references were identified. The abstracts of these 397 articles were reviewed and 24 full articles were retrieved. Of these, six articles met inclusion criteria. The characteristics of the included studies are shown in Table 1. There was complete agreement between the two reviewers regarding articles for inclusion, with a kappa statistic of 1.00. The median validity score was 4 (range 1 to 5) on a scale of 0 to 5 in which a higher number indicates higher quality. Publication bias was not seen in any of the outcomes via a visual inspection of funnel plots (data not shown).

**Table 1:** Study Characteristics

Study	Intervention	# Treated	# Controls	Patient Population	Double Blinding
					

Groothuis 1993	RSV-IGIV 750 mg/kg	81	89	Premature, CLD, CHD	No

PREVENT 1997	RSV-IGIV 750 mg/kg	250	260	Premature, CLD	Yes

Simoes 1998	RSV-IGIV 750 mg/kg	202	214	CHD	No

Subramanian 1998	Palivizumab 15 mg/kg	22	20	Premature, CLD	Yes

IMPACT 1998	Palivizumab 15 mg/kg	1002	500	Premature, CLD	Yes

Feltes 2003	Palivizumab 15 mg/kg	639	648	CHD	Yes

Of the six included studies, three utilized RSV-IGIV [11-13] (total of 533 randomized to treatment groups) and three utilized palivizumab (total of 1663 randomized to treatment groups) [14-16]. The characteristics of the included studies are shown in Table 1. One study [11] utilized high dose (750 mg/kg) and low dose (150 mg/kg) RSV-IGIV in separate treatment arms. This meta-analysis only includes data from the high dose arm. One study [14] was a palivizumab dose escalation trial and utilized separate intervention arms of 3, 10, and 15 mg/kg. This meta-analysis only includes data from the 15 mg/kg treatment arm. All of the studies gave palivizumab for only one RSV season.

### Effect on Severe RSV Disease

**Figure 2:**
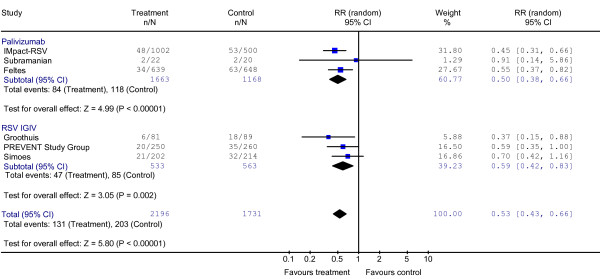
Effect of Palivizumab and RSV-IGIV on RSV Hospitalization.

Figure 2 illustrates the results of the effects of antibody prophylaxis on the primary outcome of hospitalization with confirmed RSV disease. The absolute risk of hospitalization in the control arms was 12% and overall RR for all 2,196 children who received one of the antibody products was 0.53 (95% CI 0.43, 0.66), P < 0.00001; Chi^2 ^2.97, P = 0.65, I^2 ^= 0%. When looking only at the children who received palivizumab, the RR for hospitalization was 0.50 (95% CI 0.38, 0.66), P < 0.00001; Chi^2 ^0.67, P = 0.70, I^2 ^= 0%. For the children receiving RSV-IGIV, the RR for hospitalization was 0.59 (95% CI 0.42, 0.83), P < 0.002; Chi^2 ^1.54, P = 0.46, I^2 ^= 0%. Only one study [15] specifically examined the effect of palivizumab in infants born between 32 and 35 weeks gestational age and showed an 80% reduction in RSV related hospitalization (P = 0.002). The number needed to treat with palivizumab to prevent one hospital admission was 20 (95% CI 14–33).

Table 2 illustrates the effect of prophylaxis on the secondary outcomes of ICU admission, mechanical ventilation, RSV infection, and mortality. The use of palivizumab resulted in a significant decrease in admission to the ICU (RR 0.29 (95% CI 0.14, 0.59), P = 0.0007; Chi^2 ^2.34, P = 0.13, I^2 ^= 57.3%. The number needed to treat with palivizumab to prevent one ICU admission was 20 (95% CI 9 – 30). Only one study examined the impact of palivizumab on RSV infection and did not find a significant reduction in this outcome. The use of RSV-IGIV did show a trend towards reduced RSV infection but did not reach statistical significance. There was no significant reduction in the risk of mechanical ventilation or mortality with the use of antibody prophylaxis.

**Table 2:** Primary and Secondary Outcome Measures with Prophylaxis versus Placebo

Outcome Measure	RR all Antibody Prophyaxis	RR Palivizumab	RR RSV-IGIV
RSV Hospitalization	0.53 (0.43, 0.66), P < 0.00001	0.50 (0.38, 0.66); 3 ^14,15,16^ studies, 1663 treated, P < 0.00001	0.59 (0.42, 0.83); 3 ^11,12,13^ studies, 533 treated, P = 0.002

ICU Admission	0.39 (0.21, 0.70), P = 0.002	0.29 (0.14, 0.59); 2 ^15,16^ studies, 1641 treated, P = 0.0007	0.50 (0.24, 1.04); 3^11,12,13 ^studies, 533 treated, P = 0.06

Mechanical Ventilation	0.76 (0.43, 1.36), P = 0.36	1.10 (0.20, 6.09); 2^15,16 ^studies, 1641 treated, P = 0.91	0.77 (0.33, 1.79); 3^11,12,13 ^studies, 533 treated, P = 0.55

RSV Infection	0.78 (0.60, 1.01), P = 0.06	0.45 (0.09, 2.22); 1^14 ^study, 22 treated, P = 0.33	0.79 (0.61, 1.03); 2^11,13 ^studies, 283 treated, P = 0.08

All Cause Mortality	0.95 (0.55, 1.65), P = 0.86	0.71 (0.42, 1.19); 2^15,16 ^studies, 1641 treated, P = 0.19	1.53 (0.65, 3.61); 3^11,12,13 ^studies, 533 treated, P = 0.33

Table 3 shows the results of the stratified analyses. In the stratified analyses, infants born at less than 35 weeks gestational age, and those with CLD and CHD all had a significant reduction in the risk of RSV hospitalization with children born under 35 weeks gestational age showing a trend towards the greatest benefit. The relative risk for hospitalization for premature infants receiving palivizumab was 0.2 (0.09, 0.46), P = 0.0001; however, only one study [15] provided data for this analysis. The relative risk favoring prophylaxis in premature infants receiving RSV-IGIV was 0.45 (0.18, 1.11), P = 0.08, I^2 ^= 67.9%.

**Table 3:** Stratified Analysis for RSV Hospitalization

Subgroup	RR all Antibody Prophyaxis	# of Studies	# in Treatment Groups
			

Premature <35 weeks GA[11,12,15]	0.29 (0.16, 0.54)	3	624

Chronic Lung Disease (CLD)[11,12,15]	0.55 (0.38, 0.80)	3	696

Congenital Heart Disease (CHD)[11,12,16]	0.56 (0.40, 0.79)	3	865

The overall rate of mortality was low in all groups and the majority of deaths were unrelated to RSV infection or antibody prophylaxis. The impact of antibody prophylaxis on mortality is shown in Tables 2 and 4. With the exception of Subramanian 1998 [14], all included studies provided data on duration of RSV hospitalization. However, as no measure of variance was provided for these outcomes, these data were not able to be included in this meta-analysis. However, duration of hospital stay is a surrogate marker of disease severity and thus the data is presented in Table 5.

**Table 4:** Summary of Mortality Data

Study	Deaths Treatment	Deaths Control	Notes
			

Groothuis 1993 (RSV-IGIV)	3	0	No deaths due to RSV or RSV-IGIV

PREVENT 1997 (RSV-IGIV)	5	2	No deaths due to RSV or RSV-IGIV

Simoes 1998 (RSV-IGIV)	13	13	2 deaths in treatment group & 5 in control group due to respiratory causes

Subramanan 1998 (Palivizumab)	0	1	Disseminated adenovirus

IMPACT-RSV 1998 (Palivizumab)	4	5	2 deaths in treatment group occurred during RSV hospitalization

Feltes 2003 (Palivizumab)	21	27	2 deaths in treatment group and 4 deaths in control group due to RSV

**Table 5:** Duration of Hospitalization (per 100 children)

Study	Treatment Group	Control Group
		

Groothuis 1993 (RSV-IGIV)	53.1	143.8

PREVENT 1997 (RSV-IGIV)	60	129

Simoes 1998 (RSV-IGIV)	72	107

IMPACT-RSV 1998 (Palivizumab)	36.4	62.6

Feltes 2003 (Palivizumab)	57.4	129

Adverse effects associated with palivizumab were rare and no specific adverse effect was statistically more significant in the treatment groups as compared to the control groups [15,16]. Adverse effects in RSV-IGIV recipients included fluid overload [11-13], decreases in oxygen saturation or cyanosis [11,13], fever [11,12] and respiratory distress [11,12]. In Simoes 1998 [13], there were 11 instances of unexpected cyanosis in 111 children with right to left shunts or complex cardiac defects in RSV-IGIV recipients compared to 1 such event in 83 children with similar cardiac defects in the control group (p = 0.03). In 8 of the 11 cases, the unanticipated cyanosis led to urgent surgery.

## Discussion

We found that both palivizumab and RSV-IGIV decrease the incidence of RSV hospitalization and ICU admission and their effect appears to be qualitatively similar. This study builds on a previous meta-analysis [4] through the addition of two additional randomized clinical trials. In contrast to this earlier meta-analysis which included only one palivizumab study, we have been able to separately examine the effect of palivizumab and RSV-IGIV on RSV disease severity. The numbers needed to treat with palivizumab to prevent one RSV-related hospitalization and one ICU admission were 20 (95% CI 14 – 33) and 20 (95% CI 9 – 50) respectively. Using pooled date for palivizumab and RSV-IGIV, Wang et al.[4] found that the numbers needed to treat to prevent one hospitalization and one ICU admission were 17 and 50 respectively. Our study did not find a statistically significant reduction in either the incidence of mechanical ventilation or in all cause mortality. However, due to the rare nature of these events, small differences in these outcomes might not be detectable by our meta-analytic study.

Palivizumab appeared to be safe and was not associated with any severe adverse events in any of the trials including in children with CHD. However, in post-marketing surveillance, palivizumab was very rarely associated with both anaphylactic (<1 in 100,000 patients) and hypersensitivity (1 in 1,000 patients) reactions [17]. These reactions may be more common if palivizumab is used in a second season and thus would be exceedingly unlikely to be seen in a study using the product over a single season. Conversely, RSV-IGIV appeared to be related to significant adverse events in infants and children with certain types of CHD. Additional disadvantages to RSV-IGIV include the need for intravenous access as well as the risk of infectious disease transmission and anaphylaxis that exists with any immune globulin product. Largely due to safety concerns of RSV-IGIV in children with CHD, and the availability of a safe and effective alternative prophylactic therapy, RSV-IGIV is no longer commercially available in the United States or Canada.

Palivizumab has only been evaluated in randomized controlled trials in specific groups of infants. While other groups of infants may also gain protection against severe RSV disease from this product, this has not been formally studied. Due to the high cost of palivizumab, published guidelines restrict recommendations for use to the highest risk subgroups of infants and children for whom the evidence for effectiveness is strongest. Palivizumab is currently recommended by the American Academy of Pediatrics for infants and children born at less than 32 weeks gestation, and infants born at less than 35 weeks gestation who are younger than 6 months at the beginning of RSV season and have two or more risk factors (child care attendance, school-aged siblings, exposure to environmental air pollutants, congenital abnormalities of the airways, or severe neuromuscular disease) for severe bronchiolitis. Palivizumab is also recommended for children who are 24 months of age or younger with hemodynamically significant cyanotic, acyanotic congenital heart disease, or CLD requiring medical therapy within the previous 6 months [18]. Similarly, in Canada, the Canadian Pediatric Society (CPS) recommends palivizumab for children with CLD and those born at less than 32 weeks gestation. The CPS also recommends palivizumab in the winter season be considered in those less than two years of age with hemodynamically significant CHD [19].

The cost effectiveness of palivizumab will vary by location based on the price of the drug and other health care services in a given area and the hospital and societal based cost metrics that are used. The cost that is deemed 'effective' may also differ across regions and countries. As a result, studies that attempt to determine cost effectiveness of palivizumab may not be generalizable outside of the area in which they were conducted. A recent Canadian study [20] determined the incremental cost-effectiveness of palivizumab in pre-term infants born at 32 to 35 weeks gestational age to be $20,924 per quality adjusted life year a value which is considered cost-effective in Canada. However, a very comprehensive systematic review [21] of all published cost-effectiveness studies (including in Canada, the United Kingdom, and the United States) concluded that palivizumab does not represent good value when used unselectively in preterm infants without CLD or children with CLD or CHD. Our study adds to the previous meta-analysis [4] in that it increases the precision of treatment effect estimates and increases the statistical power. Our study found the number needed to treat with palivizumab to prevent one ICU admission to be 20 in contrast to Wang et al.[4] who found that 50 infants needed to be treated with antibody product (RSV-IGIV or palivizumab). These results should prove useful in performing more precise cost-effectiveness analysis in the future.

A new monoclonal antibody, motavizumab, with increased affinity for the fusion protein of RSV has recently been developed. Motavizumab, binds to RSV fusion protein 70-fold better than palivizumab, and has a 20-fold improvement in neutralization of RSV *in vitro*. In the cotton rat model, motavizumab reduced pulmonary RSV titers to up to 100-fold lower levels than did palivizumab at equivalent concentrations. Unlike palivizumab, motavizumab also inhibited viral replication in the upper respiratory tract [20]. A large clinical trial comparing palivizumab to motavizumab showed a 26% decrease in the incidence rate of RSV hospitalization with the new product [21]. This study also demonstrated decreased RSV disease severity and decreased outpatient disease with motavizumab. Due to its expected longer serum half life, motavizumab may offer the opportunity for less than the monthly dosing required of palivizumab. However, given the expected high cost of this new product, it is unclear whether palivizumab or motavizumab is the optimal agent for RSV prophylaxis.

## Conclusion

In conclusion, the polyclonal and monoclonal RSV antibody products have the ability to significantly reduce the risk of severe RSV disease in high risk infants. This meta-analysis separately quantifies the impact of RSV-IGIV and palivizumab on various measures of severe RSV disease and builds upon a previous study that was only able to examine the pooled effect of all antibody products together. Further studies of these antibody products, and motavizumab may further define the groups that are most likely to benefit from their use.

## Competing interests

The authors declare that they have no competing interests.

## Authors' contributions

SM contributed to the design, data collection, statistical analysis, and wrote the first draft of the manuscript. BD contributed to the data collection and revisions of the draft. JB contributed to all statistical aspects and data analysis and made revisions to the manuscript. LS provided overall guidance and contributed to the design, statistical analysis, and revisions to the manuscript. All author's have read and approve the final version of the manuscript.

## Pre-publication history

The pre-publication history for this paper can be accessed here:

http://www.biomedcentral.com/1471-2334/9/106/prepub
